# Assessment of Healing Potential of *Bombyx mori* L. (Silkworm) Derivatives on Second-Degree Burns: Dose-Response and Combination Therapy Analysis

**DOI:** 10.3390/medicines12020011

**Published:** 2025-04-30

**Authors:** Evrydiki Katsikari, Alexandra Kyriaki, Andreas Vitsos, Margarita Vidali, Paschalis Harizanis, Ioannis Sfiniadakis, Maria Kostaki, Dimitra Ieronymaki, Asimina Terezaki, Georgios Ladopoulos, Chara Albani, Christina Barda, Michail Christou Rallis

**Affiliations:** 1 Division of Pharmaceutical Technology, Department of Pharmacy, National and Kapodistrian University of Athens, 15784 Athens, Greece; evryka96@gmail.com (E.K.); alex17563@gmail.com (A.K.); avitsos@pharm.uoa.gr (A.V.); margaritaki.3007@gmail.com (M.V.); marie.kstk@gmail.com (M.K.); dimitraier93@gmail.com (D.I.); lgeorge4@hotmail.com (G.L.); ch.almpani@gmail.com (C.A.); cbarda@pharm.uoa.gr (C.B.); 2 Laboratory of Sericulture & Apiculture, Agricultural University of Athens, 11855 Athens, Greece; melissa@aua.gr; 3 Pathologoanatomic Laboratory, Athens Naval Hospital, 11521 Athens, Greece; jsfiniadakis@yahoo.gr

**Keywords:** second degree burns, silkworm, *Bombyx mori*, serrapeptase, murine burn model

## Abstract

**Background/Objectives:** Burn injuries present significant treatment challenges due to the intricate nature of the healing process. *Bombyx mori* L. (silkworm) derivatives, containing healing-promoting proteins such as sericin and fibroin, as well as the anti-inflammatory enzyme serrapeptase, have shown promise as potential healing agents. This study aimed to identify the optimal dosage of silkworm body and gland extracts for burn healing, compare the selected dose’s effectiveness with that of silkworm cocoons, and assess the combined healing effects of a cocoon dressing and a silkworm body extract gel. **Methods:** An experimental model was employed using hairless SKH-hr2 female mice subjected to standardized second-degree burns. The mice received treatments with various formulations of silkworm body and gland extracts, silkworm cocoons, and a combined application of a cocoon dressing and silkworm body extract gel. **Results:** The most effective treatments were the cocoon dressing and the combination of cocoon dressing with 60% body extract gel. By Day 20, complete healing (100%) was observed in the 20% and 60% body and gland extract groups, while the cocoon and 60% gland extract groups exhibited 60% healing, significantly higher than the control group (0% healing). Wound contraction analysis showed the greatest reduction in surface area from Day 3 to Day 17 in the cocoon and 60% body extract groups (*p* < 0.05). Histopathological assessments revealed that the combination group exhibited the least tissue damage (score: 7), compared to the control (score: 10–13). **Conclusions:** The study highlights the poorly examined therapeutic potential of silkworm body and gland extracts, demonstrating their efficacy in accelerating burn healing. The effects observed by the silkworm cocoon and body extract suggests a novel and promising approach for burn wound management, warranting further clinical exploration.

## 1. Introduction

The *Bombyx mori* L., commonly known as the silkworm, is a domesticated insect belonging to the Bombycidae family within the Lepidoptera order, characterized by its complete metamorphosis life cycle [1]. This insect is central to sericulture, an agricultural practice dedicated to silkworm breeding, reproduction, pathology, and the production of cocoons [2].

The bioactive compounds of *Bombyx mori* L., including sericin, fibroin, serrapeptase, seroin, and protease inhibitors, offer a broad spectrum of therapeutic effects, highlighting their potential in various medical and health-related applications [3]. Serrapeptase, with its anti-inflammatory, analgesic, and fibrinolytic actions, has been utilized for over 40 years, especially in Japan and Europe, to reduce inflammation and pain [4]. Sericins and fibroins contribute significantly to wound healing, skin hydration, and anti-aging, thanks to their ability to bind moisture, stimulate cellular proliferation, and accelerate the regeneration of tissue [5,6]. These proteins also show antioxidant, anti-inflammatory, and antimicrobial properties, protecting against UV radiation and promoting collagen synthesis [7,8,9,10]. Seroin and protease inhibitors add to this protective spectrum by offering antimicrobial and antifungal benefits, safeguarding the silkworm, and potentially providing benefits to human health [11,12]. Collectively, these compounds from *Bombyx mori* demonstrate a multifaceted approach to health, offering benefits ranging from skin care and wound healing to dietary supplements, showcasing their versatility and potential as natural, beneficial ingredients in medical, cosmetic, and nutritional fields [13].

The concept of employing silk as a material in wound healing applications is not novel. Silk fibroin scaffolds have garnered significant attention in the literature [14] as bioactive functionalized biomaterials can be derived from specially modified silkworms [15]. There have also been research efforts focused on healing with sericin and serrapeptase [16]. The outcomes of these studies have highlighted *Bombyx mori*’s products as a source of significant bioactive components for skin healing that merit further study.

The use of *Bombyx mori* L. derivatives in burn wound healing is of growing interest due to their unique bioactive components and regenerative potential. Silk fibroin (SF), a primary structural protein of silk, has demonstrated significant therapeutic effects in skin repair and regeneration. Composed predominantly of fibroin (75–83%) and sericin (17–25%), silk also contains secondary metabolites such as flavonoids (quercetin, kaempferol), alkaloids, coumarin derivatives, and phenolic acids, which contribute to its anti-inflammatory and wound-healing properties. These bioactive constituents make *Bombyx mori*-derived materials highly biocompatible and effective in facilitating tissue repair [13].

Clinical and preclinical studies have consistently shown the benefits of silk-based dressings for burn wound healing [13]. A human study revealed that pure silk dressings accelerated re-epithelialization in superficial burns covering more than 10% of the body surface area while reducing dressing change frequency and improving patient satisfaction with scarring outcomes [17]. Similarly, experimental studies in animal models demonstrated that injectable hydrogels containing silk fibroin significantly increased wound closure, collagen deposition, and angiogenesis, all crucial factors in tissue regeneration [18]. Furthermore, the use of silk fibroin in hypertrophic scar treatment resulted in thinner, less pigmented scars with well-aligned collagen fibers, indicating its role in preventing excessive fibrotic responses [19].

The molecular mechanisms underlying silk fibroin’s regenerative properties are becoming increasingly understood. Recent studies demonstrated that silk fibroin hydrogel enhances cell proliferation, migration, and adhesion through the regulation of TLN1 expression, promoting accelerated wound healing [20]. Additionally, therapeutic dressings composed of silk fibroin have been shown to contain α-helices and β-sheets, both of which play critical roles in wound healing [17]. While α-helices improve cell proliferation and migration, β-sheet SF facilitates granulation tissue formation and re-epithelialization by enhancing extracellular matrix (ECM) protein expression (fibronectin, type III collagen), increasing matrix metalloproteinase-12 activity, and upregulating integrin β1 expression. This crosstalk between ECM proteins and cell adhesion molecules in fibroblasts highlights the ability of β-sheet SF to promote dermal tissue regeneration [17].

While extensive research has been conducted on the wound-healing properties of silkworm-derived materials, such as silk fibroin and cocoons [13], there remains a significant gap in the literature regarding the therapeutic potential of extracts obtained directly from the organism itself. Previous studies have primarily focused on silk-based dressings and hydrogels, yet the bioactivity of *Bombyx mori* body and gland extracts in wound healing remains largely unexplored. This study aimed to address this gap by conducting a comparative investigation of different extracts derived from the body and glands of *Bombyx mori*, alongside cocoon-derived components, to evaluate their effects on the healing of second-degree burns. By elucidating the wound-healing potential of these extracts, this research seeks to expand the current understanding of *Bombyx mori* bioactivity and explore novel avenues for its application in regenerative medicine.

## 2. Materials and Methods

### 2.1. Animals

In this study, 80 female SKH-hr2 mice were used. These animals were divided into 9 groups of 5 each and 5 groups of 7 each and housed separately. The SKH-hr2 mouse model was selected due to its well-documented suitability for burn healing studies. Its hairless phenotype eliminates variability in wound exposure and heat transfer, ensuring consistent burn induction and reproducible results. Additionally, the absence of fur allows for direct and uninterrupted monitoring of wound healing progression, facilitating accurate assessment of re-epithelialization, tissue remodeling, and inflammatory responses. Importantly, while murine skin differs from human skin in some structural aspects, the SKH-hr2 model shares key physiological characteristics with human epidermis, including a comparable wound healing process, making it a relevant translational model for burn injury research. Previous studies have demonstrated its efficacy in evaluating treatments that influence inflammation, collagen deposition, and angiogenesis, which are crucial factors in burn recovery [21].

The mice were sourced from the breeding facilities of the School of Pharmacy’s Small Animal Laboratory, certified under the European License Code: EL 25 BIO 06. The handling and care of the animals was in strict accordance with the European Council Directive 2010/63/EU, with ethical approval obtained from the National Peripheral Veterinary Authority’s Animal Ethics Committee under License Number 2003/5-4-2019. All procedures were conducted in adherence to ARRIVE guidelines [22].

The environmental conditions for the mice included a controlled temperature of 24 ± 1 °C, humidity of 40 ± 5%, and a 12-h light/dark cycle with lamps emitting minimal UV radiation. The animals had unrestricted access to solid food pellets (Nuevo SA, N. Artaki, Greece) and tap water.

### 2.2. Experimental Design

The study consisted of two stages. In the initial pilot part of the study, the subjects were categorized into nine cohorts, each comprising five (*n* = 5) mice, including two control cohorts. For the experimental cohorts, gels containing extracts from the silkworm body (20%, 40%, and 60% *w*/*w*) and silkworm gland (20%, 40%, and 60% *w*/*w*) were utilized, whereas patches derived from silkworm cocoons were administered.

The selection of 20%, 40%, and 60% extract concentrations was based on a systematic screening approach aimed at identifying the most effective *Bombyx mori* extracts for burn wound healing. This study represents the second phase of our research, building upon our previous findings, where we initially tested lower concentrations and progressively increased them based on observed biological activity. Through this stepwise process, the selected concentrations emerged as the most relevant for further investigation, ensuring a comprehensive evaluation of dose-dependent effects [23].

In the subsequent part, the mice were allocated into five groups, each consisting of seven mice (*n* = 7), with two serving as control groups. For the intervention groups, a gel comprising 60% *w*/*w* silkworm body extract was employed, alongside the application of silkworm cocoon patches, either in conjunction with the silkworm body extract gel or independently. All interventions are highlighted in Table 1.

### 2.3. Topical Preparations

Fifth instar silkworm larvae were obtained from the Sericulture Department of the Agricultural University of Athens shortly before they spun their cocoons. They were maintained at room temperature with a continuous supply of food (fresh mulberry leaves) for approximately four days. Enough larvae were obtained for collection of their bodies and glands. The remaining larvae were allowed to spin cocoons for about five days. The cocoons were collected when the insects had transitioned to the next biological stage, that of the pupa.

### 2.4. Silkworm Body and Gland Extraction

The silkworm body was dissected using surgical scissors to remove the glands and intestines. Subsequently, the body and glands were sectioned into smaller fragments. Water-based extracts were then prepared by immersing 10% *w*/*w* of these fragments in injectable water, avoiding light exposure, and subjecting them to continuous stirring for 24 h. Following this period, the extracts were procured via decantation and filtration.

### 2.5. Preparation of Silkworm Body Gels

Gels of 20%, 40%, and 60% concentration of extracts were prepared, along with an excipients control, by adding injectable water, 0.5% potassium sorbate (Fagron Hellas SA, Trikala, Greece), and 3% Sepigel 305 (Fagron Hellas SA Trikala, Greece), followed by homogenization (VirTis TEMPEST VirTishear, Tamil Nadu, India). The pH of the gels was finally set to 5.5 with citric acid (Fagron Hellas SA, Trikala, Greece).

### 2.6. Preparation of Cocoon Dressings

Using surgical scissors, the cocoons were longitudinally cut on one side, and the pupae inside were removed. The inner layer was cut into 2 cm × 2 cm pieces, to be applied as wound dressings.

### 2.7. Burn Wound Infliction

Initially, the animals were sedated via an intraperitoneal injection comprising a mixture of 100 mg/kg ketamine (Ketamidor, Neocell Ltd., Athens, Greece) and 7 mg/kg xylazine (Xylazine, France). Subsequently, the midpoint of the dorsum of the mice, designated for the induction of a burn, was delineated using a fine-tip marker. A bespoke metal stamp was preheated to an approximate temperature of 69 ± 2 °C in a water bath (Buchi B-480, Flawil, Switzerland). The skin over the intended area was then tautened, the stamp was extracted from the water bath, promptly blotted on a clean cloth to eliminate any residual water and positioned on the demarcated area without exerting any pressure (relying solely on its own weight) for a duration of 10 s.

### 2.8. Wound Maintenance

Dressing changes were conducted once daily. The burn area was gently dabbed with cotton soaked in injectable water, as needed, to clean the wound’s surface. The predetermined quantity of the preparation (50 mg) was then applied to the burn area. Following this, the area was covered with either a sterile square gauze or the specified cocoon dressing, depending on the treatment group (2 cm × 2 cm), and secured with adhesive tape (4 cm × 5 cm) (Fixomull^®^, Beiersdorf, Hamburg, Germany).

### 2.9. Assessment of Skin Parameters

For visual documentation, skin photographs were taken weekly with a digital Nikon camera, and an Antera 3D^®^ camera. The Antera 3D is a camera that allows for the capture of high-resolution images with the aim of evaluating treatments and processes. The Antera 3D uses an optical method combined with a complex algorithm to capture images in three dimensions. This allows for the collection of data on parameters such as the surface area, texture hemoglobin and volume of the wound.

Throughout the study, various skin characteristics were evaluated using non-invasive methods in a controlled lab environment. The investigation into the skin’s barrier to water loss involved utilizing a Tewameter TM 210 by Courage and Khazaka, based in Koln, Germany, to measure transepidermal water loss (TEWL). To assess skin moisture levels, a Corneometer CM 820 (also by Courage and Khazaka) was employed, while sebum output was gauged using a Sebumeter from the same company. The thickness of the skin was recorded using a Casio digital pachymeter.

### 2.10. Histological Assessment

At the conclusion of each experiment, the mice were euthanized, and skin biopsies were collected from the dorsal wound area for further analysis. These skin samples were then preserved in formalin, preparing them for histological examination.

The histological assessment of the mouse skin took place at the Pathology Laboratory of the Naval Hospital in Athens. The specimens were initially fixed in a 10% solution of formalin, then encapsulated in paraffin to create paraffin blocks. Thin, continuous slices were then prepared from these blocks, which underwent staining with hematoxylin and eosin (H&E) for visualization. These stained sections were scrutinized under a microscope at 100F× magnification to evaluate various parameters, including inflammation, swelling, hyperkeratosis, the thickness of the wound, and the occurrence of ulceration, necrosis, and parakeratosis, adhering to the standards set forth in Table 2.

### 2.11. Data Analysis

In cases where the data followed a normal distribution, statistical significance was assessed using parametric methods, such as the paired *t*-test and one-way ANOVA, along with post hoc LSD (least significant difference) analysis. For data not adhering to a normal distribution, non-parametric analysis methods were employed, including the Wilcoxon test and the Mann–Whitney U test. A three-phase linear regression was applied: the first regression covered the period up to Day 3, where an increase in wound surface was observed; the second regression spanned from Day 3 to Day 17, capturing the subsequent healing phase; and the third regression extended from Day 17 onward, representing the final healing phase. This method was applied consistently in both parts of the experiment to assess wound surface changes across treatment groups. The significance threshold for all analyses was set at a *p*-value of 0.05. GraphPad Prism 8.4.2 (GraphPad Software, Inc., San Diego, CA, USA) software was utilized, both for statistical analysis and the preparation of the graphs.

## 3. Results

### 3.1. General Assessment

The creation of burns resulted in second-degree burns which exhibited notable repeatability and low size variation (1.715 ± 0.4 mm^2^). Notably, in all treatment modalities during both phases of the study, the healing process appeared to surpass that of the control.

**Figure 1:**
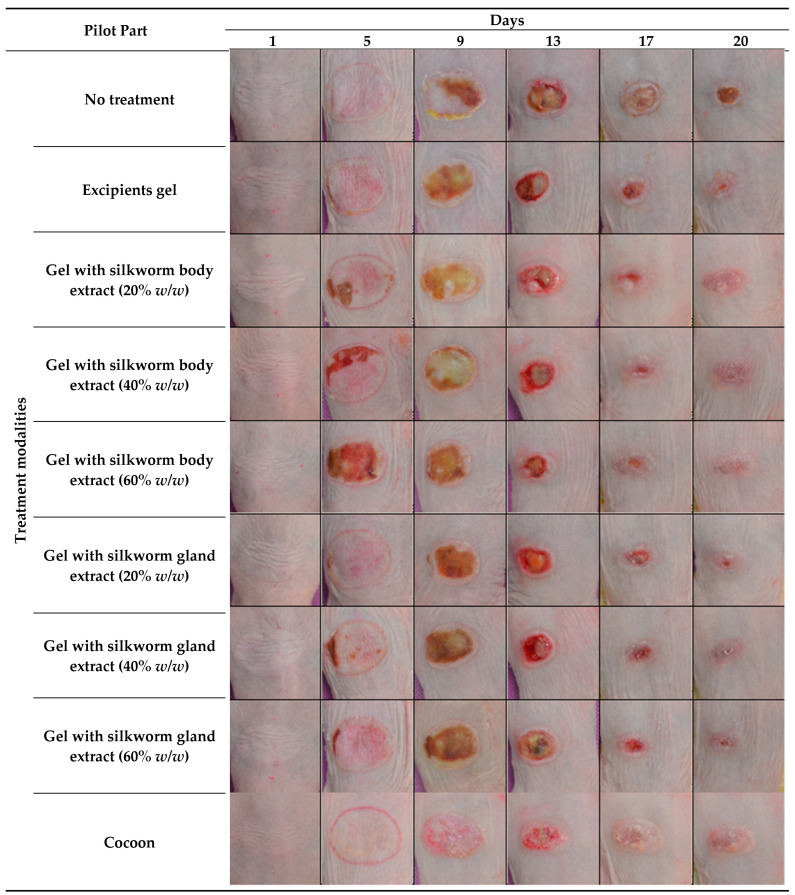
Photodocumentation of treatment modalities over time in the pilot part of the study. The figure shows representative images captured on Days 1, 5, 9, 13, 17, and 20 for each treatment modality in the pilot part of the study. The treatments include: no treatment, excipient gel, silkworm body extract gels at concentrations of 20%, 40%, and 60% *w*/*w*, silkworm gland extract gels at concentrations of 20%, 40%, and 60% *w*/*w*, and cocoon application.

During the initial stage of the investigation, the first eschar indications were observed on the third day, with this phenomenon becoming increasingly pronounced by the seventh day across all experimental groups. The eschar removal process was undertaken on the thirteenth day, resulting in a significant diminution of burn size across all groups, particularly in those administered with the 60% body extract and the cocoon dressing. By the culmination of the study, all mice within the groups receiving 20% and 60% body extracts demonstrated healing, with those treated with cocoon dressings and gland extracts at 40% and 60% concentrations showing similarly positive outcomes. Notably, the groups treated with cocoon dressings and the 60% body extract exhibited the most benign image throughout the duration of the study, characterized by a reduced eschar formation and minimal fluid exudation from the burn areas (Figure 1).

**Figure 2:**
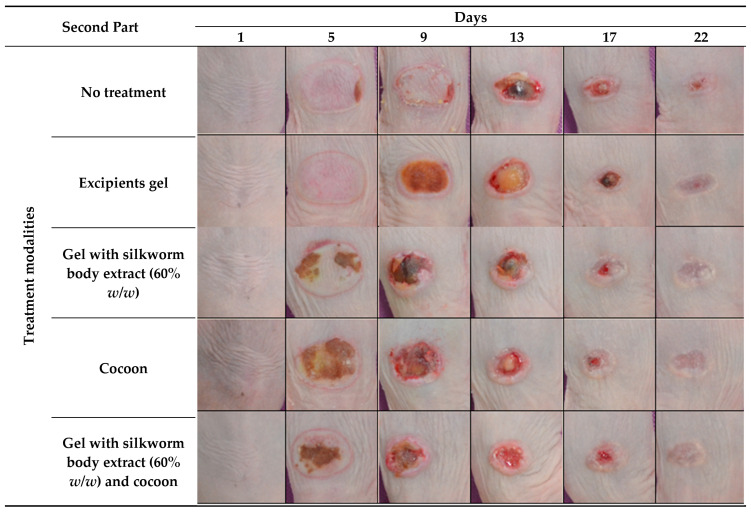
Photodocumentation of treatment modalities over time in the second part of the study. The figure displays representative images captured on Days 1, 5, 9, 13, 17, and 22 for each treatment modality in the study part of the experiment. The treatment modalities include: no treatment, excipient gel, gel with silkworm body extract (60% *w*/*w*), cocoon application, and a combination treatment of gel with silkworm body extract (60% *w*/*w*) and cocoon.

In the subsequent phase of the investigation, the appearance of eschar was notable from the outset, particularly within the group receiving the cocoon dressing, and more so in the mice subjected to the combined approach of cocoon dressing and 60% body extract, with significant intensity noted as early as the third day. For the remaining groups, the maximum amount of eschar was reached on the ninth day. Concurrently, necrotic tissue was excised from all groups on this day, facilitating a pronounced decrease in the burn sizes across the board, with the trio of intervention groups experiencing the most substantial reductions. By the experiment’s conclusion, healing was evident in six of the seven mice treated with the cocoon dressing, while the combined treatment and the 60% body extract treatment saw five out of seven mice achieve healing (Figure 2).

### 3.2. Primary Outcomes

#### 3.2.1. Percentage of Animals with Completed Healing

**Figure 3:**
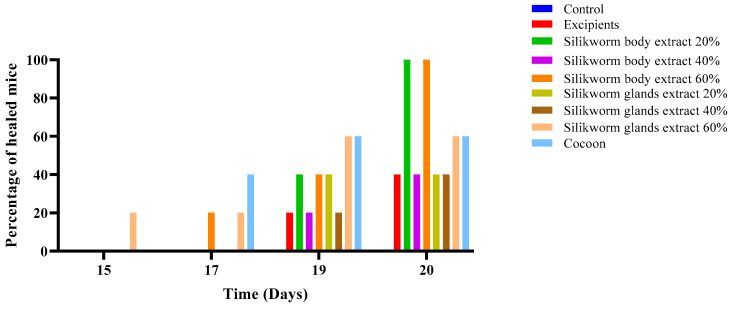
Percentage of completely healed animals over time in the pilot part of the study. The bar graph illustrates the percentage of animals that achieved complete wound healing across different treatment groups over time, specifically on Days 15, 17, 19, and 20. Treatment groups include Control, Excipients, and various formulations of silkworm body and gland extracts (20%, 40%, and 60% *w*/*w*) as well as cocoon application (*n* = 5).

During the initial phase of the study, the percentage of healed mice was calculated for each group on Days 15, 17, 19, and 20. As documented in Figure 3, the 15th day was selected as the start date for this calculation as the first healed mouse, which was from the 60% gland extract group, was observed at this time point. On the 17th day, both the 60% body extract and 60% gland extract groups demonstrated the same healing percentage, which was calculated to be 20%, while the cocoon group exhibited a 40% healed rate.

By the 19th day, the first animals in both the vehicle and the 40% body and gland extract groups had healed. On the same day, the healing percentage for the 20% and 60% body extract groups, as well as the 20% gland extract group, was 40%. Additionally, on the 19th day, the cocoon and the 60% gland extract groups each had a 60% healed rate.

On the final day of the experiment (Day 20), complete healing (100%) was observed in the 20% and 60% extract groups, followed by the cocoon and 60% gland extract groups with a healing percentage of 60%. All other groups exhibited a healing percentage of 40%, except for the control group, which did not have any healed mice throughout the duration of the study.

**Figure 4:**
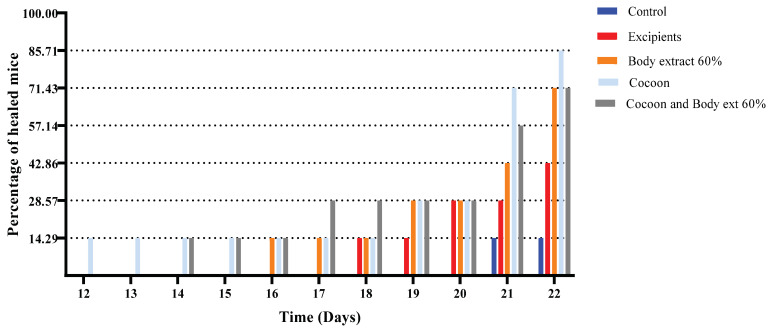
Percentage of completely healed animals over time in the study part of the experiment. The bar graph depicts the percentage of animals that achieved complete wound healing across different treatment groups over time, specifically on Days 12 to 22. Treatment groups include control, excipients, silkworm body extract gel (60% *w*/*w*), cocoon application, and a combination of cocoon and silkworm body extract gel (60% *w*/*w*) (*n* = 7).

During the days 12–22, the percentage of healed mice was calculated for each group (Figure 3 and Figure 4). The 12th day was selected as the commencement date for this calculation, as it marked the observation of the first healed mouse, which belonged to the cocoon group. By the 14th day, the first healed mouse emerged also from the combination group. On the 16th day, the first animal from the 60% body extract group healed, aligning its healing percentage to 14.29% with that of the cocoon and combination groups on the same day.

On the 17th day, an increase in the healing percentage of the combination group was observed, reaching 28.57%, with the rest of the groups maintaining the same levels as on the 16th day. The 18th day saw similar results, with an additional healed mouse from the vehicle group. On the 19th day, an increase in the healing percentages of the cocoon and 60% body extract groups was noted, both reaching 28.57% and matching the healing percentage of the combination group. By the 20th day, the vehicle group had also reached these levels, while the control group remained without any healed mice.

By the 21st day, the healing percentage of the cocoon group reached 71.43%, with the combination and 60% body extract groups following at 57.14% and 42.86%, respectively. On the same day, two healed mice were found in the vehicle group, while the first healed animal emerged from the control group.

On the final day of the experiment (Day 22), a healing percentage of 85.71% was observed for the cocoon group and 71.43% for both the combination and 60% body extract groups. The excipients group had three healed mice, while the control group remained with one healed mouse.

#### 3.2.2. Wound Surface

**Figure 5:**
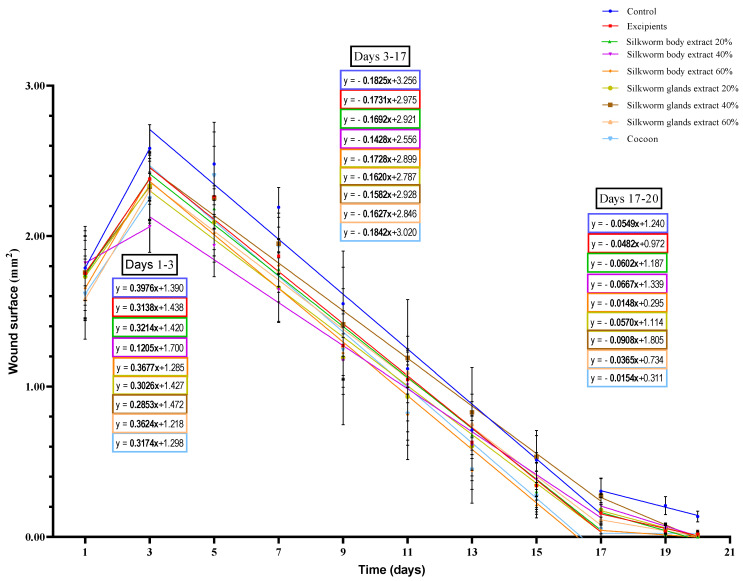
Linear regression analysis of wound surface change over time in the pilot part of the study (*n* = 5). The graph displays wound surface area (in mm^2^) over time (in days) for different treatment groups in the pilot study, including control, excipients, silkworm body and gland extract gels at various concentrations, and cocoon. Linear regression lines are shown for three distinct phases of healing: Days 1–3, Days 3–17, and Days 17–20. The slope of each regression line represents the rate of wound healing (surface area reduction) for each treatment within each phase.

Initially, up to the third day of the experiment, an observable increase in wound surface area was noted across all groups, with this increase being statistically significant in most cases (Figure 5).

In the study’s initial phase, the escalation in wound surface area followed a particular order when ranked by group, in descending order of magnitude: the control group exhibited the most significant increase, followed by the 60% silkworm body extract group, then the 60% silk gland extract group, with subsequent decreases observed in the 20% silkworm body, cocoon, vehicle, 20% silk gland, 40% silk gland, and the 40% silkworm body extract groups.

Additionally, the analysis reveals two distinct rates of reduction in the burn surface area across all groups from third day to twentieth day. The first rate, observed from the third to seventeenth day, suggests a sequence in descending order as follows: cocoon, control, 60% silkworm body extract vehicle, 20% silkworm body extract, 60% silk gland extract, 20% silk gland extract, 40% silk gland extract, and 40% silkworm body extract. The second rate, from the seventeenth to twentieth day, indicates a different order: 40% silk gland extract leading, followed by 40% silkworm body extract, 20% silkworm body extract, 20% silk gland extract, control, vehicle, 60% silk gland extract, cocoon, and finally, 60% silkworm body extract.

On the third day of the first experiment, the burn surface area for the group treated with 40% silkworm body extract was found to be significantly smaller in comparison to all other groups, apart from the cocoon group. On this day, statistically significant differences were observed between the control group and the cocoon group, as well as between the control group and the three groups treated with silk gland extracts at varying concentrations.

By the fifth day, the burn surface area in the control group proved to be significantly larger than those observed in the groups treated with the three concentrations of silkworm body extract and the 20% and 60% silk gland extracts. On the same day, the burn surface area for the 40% body extract group was significantly smaller compared to the excipients, the 40% silk gland extract group, and the cocoon group.

On the seventh day, the burn surface area of the control group was significantly larger compared to all other groups. On the same day, the burn surface areas of the 40% body extract group and the 20% gland extract group were significantly smaller in comparison to the cocoon group and the 40% gland extract group.

By the ninth day, the burn surface area for the control group was found to be significantly larger than those of the 40% body extract group and the 20% gland extract group. On the eleventh day, the cocoon group and the 60% body extract group exhibited significantly reduced burn surface areas compared to the 40% gland extract group.

On the thirteenth day, the burn surface areas of the cocoon group and the 60% body extract group were significantly smaller than that observed in the control group and the 40% and 60% gland extract groups.

By the fifteenth day, the burn surface area for the control group was significantly larger compared to the 20% and 60% body extract groups, as well as the cocoon group. On the same day, the 40% gland extract group exhibited a significantly increased burn surface area compared to the excipients, the 20% and 60% body extract groups, the 60% gland extract group, and the cocoon group.

On the seventeenth day, the burn surface area for the control group was significantly larger than those of the excipients, the 20% and 60% body and gland extracts groups, and the cocoon group. On the same day, the burn surface area of the 60% body extract group was significantly smaller compared to the excipients, the 20% and 40% body and gland extracts groups. The cocoon group also showed similar results, apart from the comparison with the excipients, which was not statistically significant. Furthermore, significant differences were noted between the 40% gland extract group and the excipients and the 60% gland extract group.

On the nineteenth and twentieth days, the burn surface area for the control group was significantly larger compared to all other groups.

**Figure 6:**
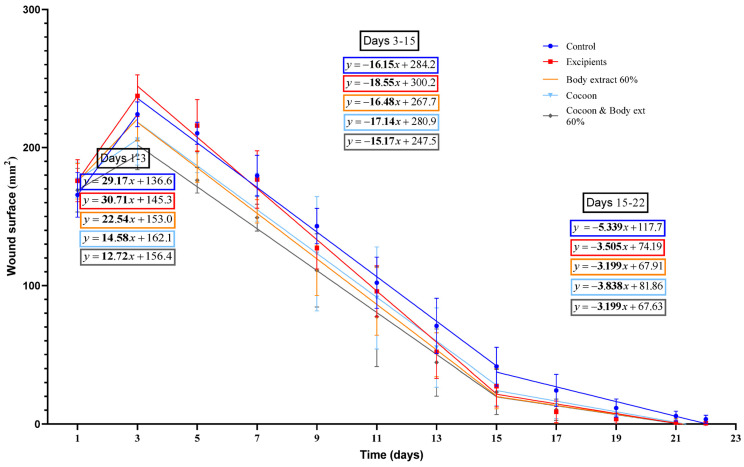
Linear regression analysis of wound surface change over time in the study part of the experiment (*n* = 7). The graph illustrates the wound surface area (in mm^2^) over time (in days) for different treatment groups in the study part of the experiment, including control, excipients, silkworm body extract gel (60% *w*/*w*), cocoon, and a combination of cocoon and silkworm body extract (60% *w*/*w*). Linear regression lines are presented for three phases: Days 1–3 (wound surface increase phase), Days 3–15 (initial healing phase), and Days 15–22 (final healing phase).

During the study’s second phase (Figure 6), the initial increase in wound surface area up to third day exhibited a distinct pattern, ranging from the highest to the lowest as follows: vehicle, control, 60% silkworm body extract, cocoon, and the combination of cocoon and 60% silkworm body extract groups.

Moreover, from Day 3 to Day 22, two differentiated rates of burn surface area reduction were identified. The first, spanning from the third to fifteenth, ranked in descending order, were as follows: Vehicle, cocoon, 60% silkworm body extract, control, and the combination of cocoon and 60% silkworm body extract. The subsequent rate, from Day 15 to 22, presented a different order, as follows: control, cocoon, vehicle, and then both the 60% silkworm body extract and its combination with cocoon equally.

The combination group (cocoon and 60% body extract) in the second experiment, the burn surface area on Day 3 was significantly smaller compared to that of the control group, the excipients, and the 60% body extract group. Statistically significant differences were also observed between the cocoon alone and the control group, between the cocoon and the excipients, as well as between the 60% body extract group and the excipients.

On Day 5, the burn surface area for both the control group and the excipients was significantly larger compared to the combination group (cocoon and 60% body extract), the cocoon alone, and the 60% body extract group. A statistically significant difference was noted between the cocoon and the combination group on the same day.

By Day 7, the burn surface area for the control group and the excipients was significantly larger compared to that of the combination group (cocoon and 60% body extract) and the 60% body extract group. On Day 9, it was observed that only the control group had a wound surface area significantly larger than that of the combination group and the 60% body extract group.

On Days 13 and 15, a comparison between the burn surface area of the control group and that of the combination group (cocoon and 60% body extract) showed a statistically significant difference, with the combination group having a smaller surface area on both days.

On Days 17, 19, and 22, the burn surface area for the control group was significantly larger compared to that of the combination group (cocoon and 60% body extract), the cocoon alone, the 60% body extract group, and the excipients. The same results were obtained on Day 21, with the exception that the statistically significant difference between the control group and the 60% extract group was not observed.

#### 3.2.3. Histopathological Assessment

**Figure 7:**
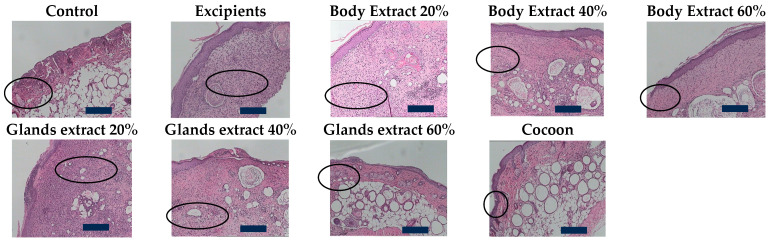
Histopathological assessment of burn wounds at 20 days post-burn (pilot part) at 100× magnification (scale bar = 100 µm)**.** The figure displays representative histological images of hematoxylin and eosin-stained sections (5 μm thickness) of burn wounds from the pilot study, captured at 100× magnification. These images correspond to different treatment groups and provide a detailed view of the inflammatory response, edema, hyperkeratosis, ulceration, and other tissue characteristics at 20 days post-burn. The control and excipient groups exhibit severe inflammation with dense polymorphonuclear leukocyte infiltration, while the cocoon group shows mild inflammation with limited tissue damage. The histopathological assessment revealed that treatment with silkworm extracts, particularly in the cocoon group, led to reduced inflammation and edema compared to the control. The areas of inflammation are indicated by ‘
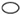
’.

During pilot part, in the control group, severe inflammation was evidenced, characterized by a significant presence of polymorphonuclear leukocytes across the entire thickness of the dermis. Additionally, marked edema and limited moderate hyperkeratosis with the presence of ulceration and necrosis were noted. The overall damage was scored at 13 points. Similar dense inflammatory infiltrates were found in the vehicle group, with the extent of the damage being total, accompanied by severe edema and heavy, widespread hyperkeratosis. The total damage in this group was scored at 12 points (Figure 7, Table 3).

For the groups treated with the three different concentrations of silkworm body extract, the observed inflammation and resultant edema were of moderate intensity and extended through the entire thickness of the dermis. Hyperkeratosis was widespread and mild, except in the group treated with the 20% body extract, where it was of moderate intensity. Additionally, in the group treated with 60% body extract concentration, scar formation was observed. The overall damage for the 20% body extract group was scored at 9 points, while for the other two body extract groups, it was scored at 8 points (Figure 7, Table 3).

The groups treated with the three concentrations of gland extracts exhibited a particularly high number of polymorphonuclear leukocytes throughout the dermis and severe edema, accompanied by intense inflammation and the presence of necrosis and ulcerations. The observed hyperkeratosis showed some variation among these three groups. In the case of the 20% gland extract, it was severe but localized. For the 40% gland extract concentration, it was both severe and extensive, whereas for the 60% concentration, the hyperkeratosis was moderate and widespread. The overall damage for the 20% and 40% gland extracts was scored at 14 points, while for the 60% extract, it was scored at 13 points (Figure 7, Table 3).

Finally, the cocoon group exhibited mild inflammation with the presence of a few polymorphonuclear leukocytes, accompanied by mild edema. The damage thickness was superficial, and the hyperkeratosis was moderate and extensive, leading to an overall score of 5 points (Figure 7, Table 3).

**Figure 8:**
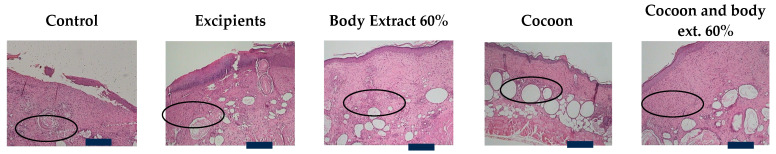
Histopathological assessment of burn wounds at 20 days post-burn (second study part) at 100× magnification (Scale bar 100 μm). The figure presents representative histological images of hematoxylin and eosin-stained sections (5 μm thickness) of burn wounds from the study part of the experiment, captured at 100× magnification. These images correspond to different treatment groups and reveal details of inflammatory response, edema, hyperkeratosis, ulceration, and other tissue characteristics at 20 days post-burn. The control and excipient groups demonstrate severe inflammation and edema, with extensive tissue damage across the entire dermal thickness. In contrast, the cocoon and combination treatment groups show moderate inflammation and edema with limited tissue damage. The areas of inflammation are indicated by ‘
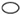
’.

In the second experiment of the study, the control group exhibited severe inflammation combined with moderate edema, with the damage extending across the entire thickness of the dermis. Additionally, mild and limited hyperkeratosis was observed alongside ulceration. The total damage in this group was scored at 10 points (Figure 8, Table 4).

Similarly, dense inflammatory infiltrations were found in the vehicle group, with the damage being total in thickness, accompanied by severe edema and mild, extensive hyperkeratosis. Notably, the presence of an inflammatory exudate was characteristic. Ulceration, parakeratosis, and the formation of a subdermal blister, hemorrhagic in nature (with the presence of red blood cells), were also observed. The total damage in this group was scored at 12 points (Figure 8, Table 4).

For the group treated with 60% silkworm body extract, the observed inflammation and resulting edema were of moderate intensity, covering the entire thickness of the dermis. Hyperkeratosis was mild and widespread, resulting in a total damage score of 8 points (Figure 8, Table 4).

The cocoon group displayed dense inflammation with a band-like distribution in the lower dermis and the presence of sparse and scattered inflammatory cells in the upper dermis, with the total thickness of the damage being significant. The edema was characterized as moderate, and the hyperkeratosis was mild and extensive. The overall damage in this group was scored at 8 points (Figure 8, Table 4).

Finally, for the combination group, the observed inflammation and resulting edema were moderate. The thickness of the damage was also moderate, and the hyperkeratosis was mild and limited. Consequently, a score of 7 points was assigned (Figure 8, Table 4).

### 3.3. Secondary Outcomes

#### 3.3.1. Transepidermal Water Loss (TEWL)

**Figure 9:**
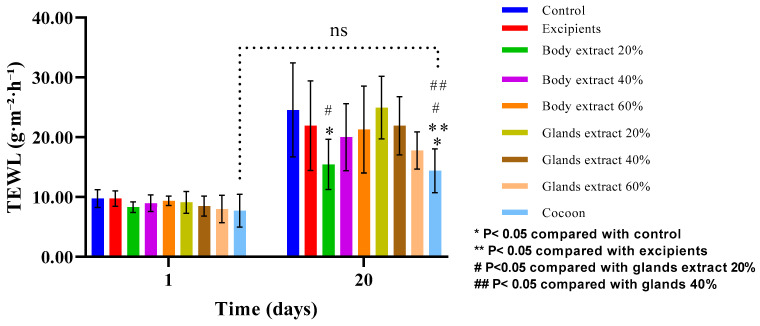
Transepidermal water loss (TEWL) measurements over time across different treatment modalities within the pilot part of the study. The bar graph shows transepidermal water loss (TEWL) measurements (g/m^2^/h) at Days 1 and 20 for various treatment groups (*n* = 5), including control, excipients, silkworm body extracts (20%, 40%, and 60% *w*/*w*), silkworm gland extracts (20%, 40%, and 60% *w*/*w*), and cocoon. Statistically significant differences are indicated: * *p* < 0.05 compared with control, ** *p* < 0.05 compared with excipients, # *p* < 0.05 compared with glands extract 20%, and ## *p* < 0.05 compared with glands extract 40%. The “ns” label indicates no significant difference between certain groups over time. Error bars represent standard deviations.

In the pilot experiment, the paired *t*-test revealed statistically significant variations across various groups of TEWL measurements between the onset and conclusion of the study. Specifically, the control, 20% body extract, 40% body extract, 60% body extract, 20% gland extract, 40% gland extract, and 60% gland extract groups all exhibited an increase in values by the study’s end, signifying a failure to revert to baseline levels. Conversely, the excipients and cocoon groups did not demonstrate statistically significant changes, suggesting a potential normalization to baseline values (Figure 9).

Further analysis using one-way ANOVA indicated no significant disparities among the groups at the baseline before the induction of the burn injury. Nevertheless, significant differences emerged by the study’s end, highlighting distinct outcomes across treatments. Notably, comparisons between the control and 20% body extract, control and cocoon, excipients and cocoon, 20% body extract and 20% gland extract, 20% gland extract and cocoon, 40% gland extract and cocoon, as well as between 20% gland extract and 60% gland extract groups, all yielded statistically significant differences.

**Figure 10:**
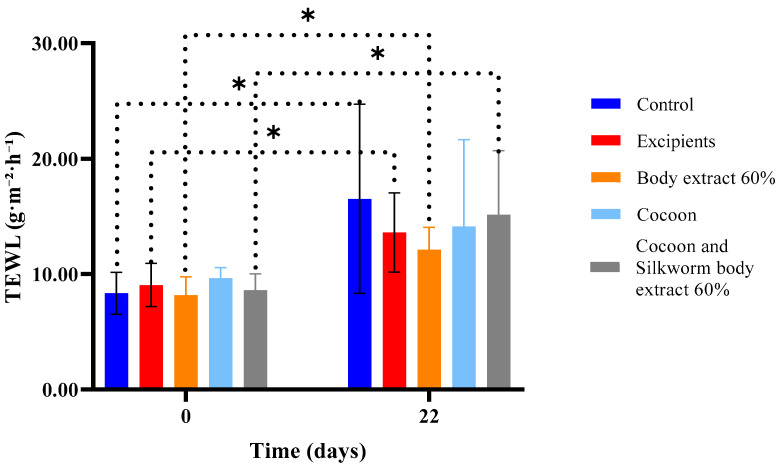
Transepidermal water loss (TEWL) measurements over time across different treatment groups in the study part of the experiment (*n* = 7). The bar graph illustrates transepidermal water loss (TEWL) measurements (g/m^2^/h) at Days 0 and 22 for various treatment groups, including control, excipients, silkworm body extract (60% *w*/*w*), cocoon, and a combination of cocoon and silkworm body extract (60% *w*/*w*). Statistically significant differences between groups are marked with an asterisk (* *p* < 0.05). The dotted lines indicate significant comparisons across time points and treatment groups, reflecting the impact of treatments on skin barrier function. Error bars represent standard deviations.

Regarding the second experiment (Figure 10), the results depicted in the graph lead to the conclusion that, on the last day of the experiment, all groups exhibited increased values of transepidermal water loss. The only group that did not show a statistically significant difference in this parameter between Day 0 and Day 22 was the one treated with the cocoon as a dressing.

#### 3.3.2. Hydration

**Figure 11:**
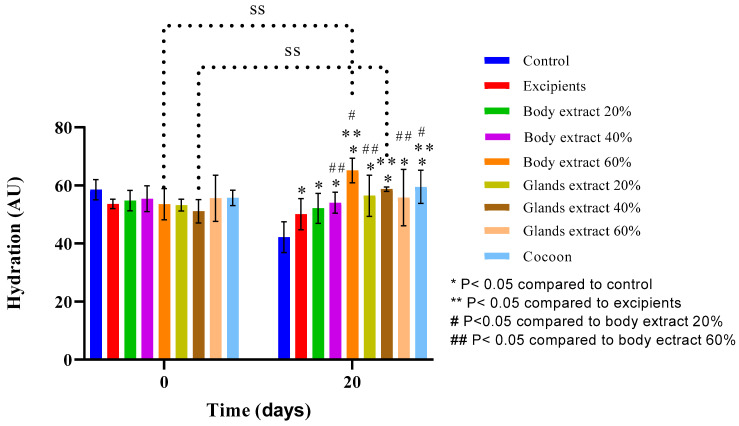
Skin hydration measurements over time across different treatment groups in the pilot part of the study (*n* = 5). The bar graph presents skin hydration levels (AU) at Days 0 and 20 for various treatment groups, including control, excipients, silkworm body extracts (20%, 40%, and 60% *w*/*w*), silkworm gland extracts (20%, 40%, and 60% *w*/*w*), and cocoon. Statistically significant differences are indicated: * *p* < 0.05 compared with control, ** *p* < 0.05 compared with excipients, # *p* < 0.05 compared with body extract 20%, and ## *p* < 0.05 compared with body extract 60%. “SS” denotes significant differences over time within groups. Error bars represent standard deviations, showing variations in hydration levels between treatment groups.

In the initial experiment (Figure 11), analysis indicated that, for all groups other than the control, 60% body extract, and 40% gland extract groups, no statistically significant differences in hydration levels were found from the start to the end of the experiment. Notably, hydration was significantly reduced in the control group by Day 20, but the 60% body extract and 40% gland extract groups experienced a significant increase in hydration. A statistically significant variation was found between the control group and the 40% gland extract group at the beginning, suggesting initial differences in hydration levels between these groups.

By Day 20, hydration in the control group was significantly lower than in all other groups, marking a notable decline in hydration within this group over the duration of the experiment. On the same day, the difference in hydration between the 40% gland extract group and the excipients was statistically significant, underlining the superior hydrating effect of the 40% gland extract. Furthermore, on Day 20, the 60% body extract group’s hydration was significantly higher compared to the excipients, the 20% and 40% body extract groups, and the 20% and 60% gland extract groups. Additionally, the hydration level of the cocoon group on Day 20 was significantly greater in comparison to that of the excipients and the 20% body extract group, demonstrating the cocoon’s efficacy in enhancing skin moisture above these treatments. These findings highlight the differential impact of various treatments on skin hydration, with certain formulations, notably the 60% body extract and the cocoon, showing significant potential for improving skin moisture retention.

**Figure 12:**
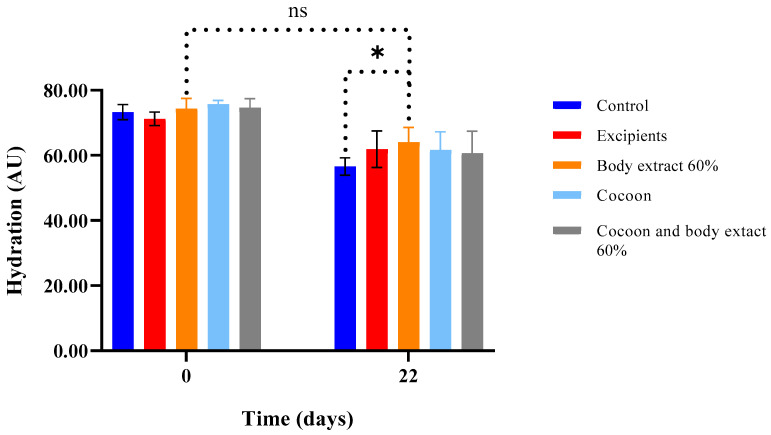
Skin hydration measurements over time across different treatment groups in the study part of the experiment (*n* = 7). The bar graph shows skin hydration levels (AU) at Days 0 and 22 for various treatment groups, including control, excipients, silkworm body extract (60% *w*/*w*), cocoon, and a combination of cocoon and silkworm body extract (60% *w*/*w*). A statistically significant difference is marked with an asterisk (* *p* < 0.05), while “ns” indicates no significant difference between certain groups over time. Error bars represent standard deviations, illustrating hydration variability within each group.

In the second experiment of the study, as illustrated in Figure 12, it was concluded that on the final day of the experiment, all groups exhibited decreased hydration levels compared to the start point. Controversy, the group treated with the 60% silkworm body extract was the only one that did not show a statistically significant difference in this. Furthermore, a statistically significant difference was observed between the group treated with the 60% silkworm body extract and the control group on Day 22.

#### 3.3.3. Sebum

**Figure 13:**
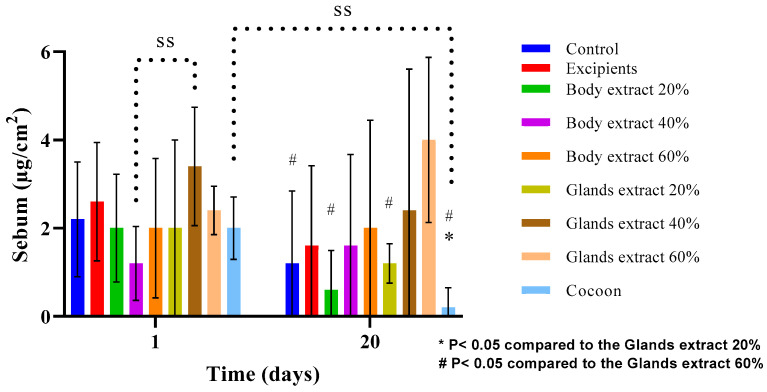
Sebum measurements over time across different treatment groups in the pilot part of the study (*n* = 5). The bar graph presents sebum levels (µg/cm^2^) at Days 0 and 20 for various treatment groups, including control, excipients, silkworm body extracts (20%, 40%, and 60% *w*/*w*), silkworm gland extracts (20%, 40%, and 60% *w*/*w*), and cocoon. Statistically significant differences are marked as follows: * *p* < 0.05 compared with the glands extract 20%, and # *p* < 0.05 compared with the glands extract 60%. “SS” indicates significant differences over time within specific groups. Error bars represent standard deviations, showing variability in sebum levels between treatment groups.

In the first experiment of the study (Figure 13), analysis revealed no statistically significant differences across any of the groups in terms of sebum measurements. However, it was observed that sebum levels tended to decrease on the last day compared to the first day across all groups, with the exceptions being the 40% body extract and the 60% gland extract groups, which showed an upward trend, and the 60% body extract group, where levels remained consistent. This indicates that, while overall sebum production may decrease over time with the application of various treatments, specific formulations like the 40% body extract and the 60% gland extract can potentially stimulate sebum production or maintain it at initial levels.

Furthermore, there were no statistically significant differences between the groups, except for a notable distinction between the 40% body extract and the 40% gland extract groups, indicating an initial significant variance in sebum levels between these two treatments. As the experiment progressed, statistically significant differences were observed among several groups, highlighting the distinct impact of certain treatments on sebum production. Notably, significant differences were found between the 20% gland extract and the cocoon, the 60% gland extract and the 20% body extract, the 60% gland extract and the 20% gland extract, the 60% gland extract and the control group, and the 60% gland extract and the cocoon. These results suggest that treatments can have varied effects on sebum levels, with the 60% gland extract showing a particularly significant impact compared to other treatments, affecting sebum production in a notable manner.

**Figure 14:**
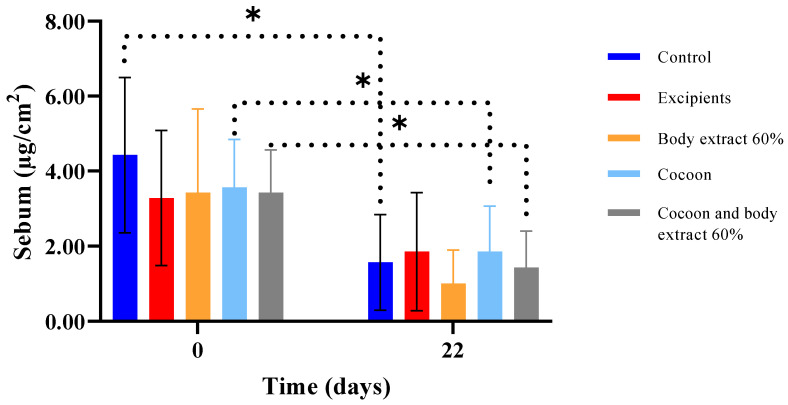
Sebum measurements over time across different treatment groups in the study part of the experiment (*n* = 7). The bar graph displays sebum levels (µg/cm^2^) at Days 0 and 22 for various treatment groups, including control, excipients, silkworm body extract (60% *w*/*w*), cocoon, and a combination of cocoon and silkworm body extract (60% *w*/*w*). Statistically significant differences are indicated by an asterisk (* *p* < 0.05). Dotted lines represent significant comparisons between groups and time points. Error bars denote standard deviations, illustrating the variability in sebum levels within each group.

In the second experiment, as illustrated in Figure 14, it was concluded that all groups exhibited reduced sebum levels by the last day of the study. The only groups that did not show a statistically significant difference in this parameter between Day 0 and Day 22 were the excipients and the 60% silkworm body extract. Furthermore, comparisons between all groups were conducted on Day 0 and Day 22 did not reveal any statistically significant differences.

#### 3.3.4. Skinfold Thickness

**Figure 15:**
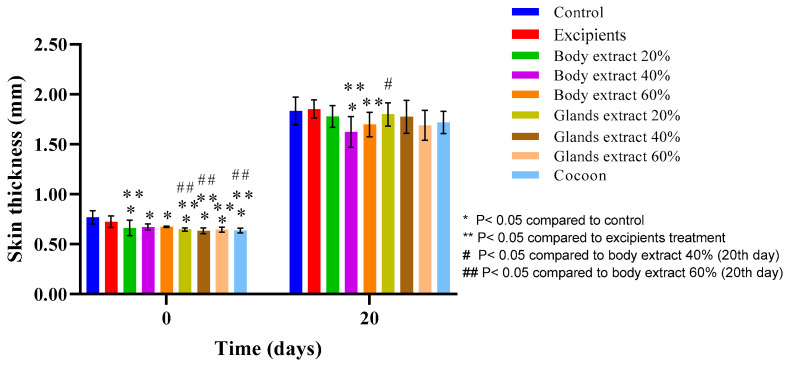
Skin thickness measurements over time across different treatment groups in the pilot part of the study (*n* = 5). The bar graph illustrates skin thickness (mm) at Days 0 and 20 for various treatment groups, including control, excipients, silkworm body extracts (20%, 40%, and 60% *w*/*w*), silkworm gland extracts (20%, 40%, and 60% *w*/*w*), and cocoon. Statistically significant differences are denoted as follows: * *p* < 0.05 compared with control, ** *p* < 0.05 compared with excipients, # *p* < 0.05 compared with body extract 40% (20th day), and ## *p* < 0.05 compared with body extract 60% (Day 20). The measurements capture variations in skin thickness between groups, with error bars representing standard deviations.

In the pilot experiment of the study (Figure 15), an upward trend in skin thickness was observed across all groups on the last day compared to the initial measurements. The paired *t*-test indicated statistically significant differences in all groups, except for the excipients and the 60% body extract group.

According to the one-way ANOVA test, significant differences in skin thickness values were observed on the first day before the burn among the following groups: control compared with each of the 20%, 40%, and 60% body extract groups, all gland extract groups (20%, 40%, 60%), and the cocoon group, as well as between the excipients and each of the mentioned groups. This indicates initial variations in skin thickness among the different treatment groups. On Day 20, significant differences were noted between the control and the 40% body extract group, excipients and the 40% and 60% body extract groups, and the 40% body extract group and the 20% gland extract group.

**Figure 16:**
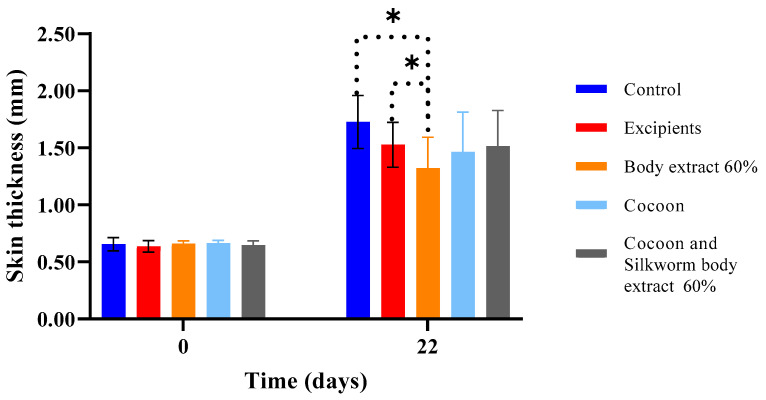
Skin thickness measurements over time across different treatment groups in the study part of the experiment (*n* = 7). The bar graph shows skin thickness (mm) at Days 0 and 22 for various treatment groups, including control, excipients, silkworm body extract (60% *w*/*w*), cocoon, and a combination of cocoon and silkworm body extract (60% *w*/*w*). Statistically significant differences are marked with an asterisk (* *p* < 0.05), indicating notable comparisons between groups. Error bars represent standard deviations, highlighting variability in skin thickness within each group.

In the second experiment of the study (Figure 16), as demonstrated in the graph, it was concluded that by the final day, all groups exhibited statistically significant increases in skin thickness compared to the beginning. Notably, significant differences emerged between the group treated with 60% silkworm body extract and both the control and excipients groups on Day 22.

## 4. Discussion

The present study aimed to examine the action of three different silk moth products (silkworm body, glands, and cocoon) at different doses. One of the primary objectives of this research was to highlight the body and glands of the silk moth as potential sources of bioactive ingredients for burn healing. For this purpose, a burn model in experimental animals (SKH-hr2 female mice) was utilized. The study was conducted in two phases, where the products with the best outcome initially, (cocoon and 60% body extract) were further tested on more subjects, both individually and in combination.

As per primary outcomes [24], based on initial results, complete healing was observed on the final day for groups treated with body extracts at concentrations of 20% and 60%, followed closely by groups treated with cocoon and gland extracts at 60%, each with three healed mice. In the subsequent phase of the study, on Day 19, the healing rates of the three treatments were at comparable levels. By Day 21, a significant increase in the number of healed muscles was observed in the group treated with cocoon extracts, as well as a substantial improvement in the healing rate of the group receiving a combination therapy. Ultimately, the cocoon extract group exhibited the highest healing rate, with the other two treatments being equally effective. The control groups’ healing rates remained at lower levels.

In all experimental groups, there was a noticeable enlargement of the burn surface area on Day 3 compared to initial measurements, a phenomenon potentially attributable to the well-documented process of burn wound conversion [25]. Subsequently, formation of eschar was observed, with a reduced quantity noted in groups treated with cocoon extracts and 60% silkworm body extract. By the conclusion of the experiment, healing was evident in all mice within the groups administered 20% and 60% silkworm body extracts, followed closely by those receiving cocoon and silk gland extracts at 40% and 60% concentrations. This suggests that specific compounds present in the silkworm body, along with those identified in the cocoon and silk glands, may play a significant role in enhancing the rate of healing in the treated groups. Indeed, it is well-known that sericin and fibroin have proven healing properties [26,27].

Multiple comparisons between groups on daily measurements were undertaken regarding the burn surface area uncovered two distinct trends during the initial and latter halves of the experiment. In the first period (Days 1–9), the 40% body extract and 20% gland extract groups exhibited the most effective healing. Nevertheless, the formation of eschar during these initial days occurred at variable rates and quantities, thereby compromising the reliability of comparisons between the therapeutic groups.

According to the histopathological results, initially animals treated with cocoon demonstrated the best histopathological profile, with the observed damage graded at 5 units. The inflammation and edema were mild, and the depth of the damage was superficial. Such healing action also has previously been attributed to the anti-inflammatory and wound healing properties of sericin and fibroin [26,27]. The next best performing groups were those treated with body extracts at concentrations of 40% and 60%, which were scored at 8 units. In these groups, the inflammation and edema were of moderate severity, while the damage extended throughout the thickness of the dermis. The satisfactory results may have been aided by the well-known anti-inflammatory action of serrapeptase [28]. The group treated with body extract at a 20% concentration was scored slightly higher, revealing that an increase in dosage might yield better results in healing burns. Notably, the gland extract groups, especially at the lower dosages, received particularly high histopathological scores, exceeding those of the control groups. This reveals an internal damage greater than evidenced, albeit slightly less for the 60% gland extract; this phenomenon may possibly be attributed to seroin activity [29], which is produced in the glands.

In the second experiment, the combination group (cocoon and 60% body extract) exhibited the best histopathological picture, with the observed damage graded at 7 units. The inflammation, edema, and depth of the damage were of moderate severity. It seems that the two silk products may act slightly better in synergy, according to the histopathological findings. The immediate next best groups were those treated with 60% body extract and cocoon, each scored at 8 units. In these groups, the inflammation and edema were of moderate severity, and the damage extended through the entire thickness of the dermis.

Regarding the quality of healing observed through the secondary outcomes of this study, the following observations can be made. Within the first experiment, the transepidermal water loss (TEWL) appears increased for all groups on the final day of the experiment, a predictable outcome as burns disrupts the normal functioning of the epidermal barrier due to the destruction of the keratin layer [30], which, as expected, did not return to normal values [31]. However, the only group that did not show a statistically significant difference between the first and the last day of the experiment in terms of TEWL was the one treated with cocoon. This suggests that the TEWL values for this treatment group tend to return to normal levels. Additionally, hydration results indicate a reduction for almost all experimental groups on the last day of the experiment compared to the initial measurement. This reduction could be attributed both to the destruction of the epidermis, thus decreasing the skin’s ability to retain water and hydrate, and to the presence of edema or inflammation [32]. This reduction was statistically significant only for the control group, while in the other groups, where the reduction is not statistically significant, hydration tends to return to initial levels. For the groups treated with 60% body extract and 40% gland extract, a statistically significant increase in hydration was observed. In the case of the 60% gland extract group, as confirmed by histopathological data, the increased hydration is due to increased inflammation and edema. Conversely, for the 60% body extract group, in line with histopathological data, there is no evidence of significant inflammation in the area, and the increased hydration compared to the control group can be linked to the optimal functioning of post-traumatic tissue. Similar results were obtained from the second experiment, where, again, the group treated with the cocoon as a topical dressing experienced a non-statistically significant increase in transepidermal water loss (TEWL), while the other groups showed statistically significant TEWL. Meanwhile, the body extract did not exhibit the statistically significant reduction in hydration observed in the other interventions.

It is well established that burn injuries lead to thickening of the skin due to scar formation [33]. Thus, on the final day of the experiment, increased thickness values were observed across all groups, which were statistically significant compared to the first day of the experiment. Comparing between treatments, statistically significant results on the last day, which include the significantly reduced thickness for the groups treated with 40% and 60% body extract in comparison to the excipients group, as well as the significant difference between the 20% gland extract and 40% body extract groups. This indicates that, in terms of skin thickness, the 40% and 60% body extract groups demonstrated a smaller increase in this parameter. This outcome is consistent with the histopathological results, which showed that the hyperkeratosis observed in these treatments was milder compared to the other groups. This may be attributed to the anti-inflammatory action of serrapeptase present in the body extract (in higher concentrations in the higher dose extracts), resulting in diminished scar tissue formation during healing process. Additionally, during the second experiment, the statistically significant differences observed on the final day between the control groups (untreated and excipients) and the 60% body extract group are noteworthy, as the treatment may have limited the thickening of the new epidermis. However, in the case of the combination therapy, the thickness did not significantly differ from that of the control groups on the last day, which may imply that the cocoon in combination with the extract did not act with the same intensity in limiting the increase in skin thickness compared to the individual treatment (60% body extract).

The results of our study on the effectiveness of the silkworm cocoon and 60% body extract in wound healing may be attributed to the synergistic actions of bioactive compounds present in these extracts. Key components of the cocoon, such as sericin and fibroin, are known to possess wound-healing and anti-inflammatory properties, which could participate in tissue regeneration and reduce inflammation at the site of injury. Additionally, the serrapeptase enzyme found in silkworm body extracts might further enhance healing by promoting the breakdown of fibrin and decreasing inflammatory responses. It is worth noting that the observed effectiveness could also be due to the combined impact of these components working together to accelerate the healing process.

Although these findings are promising, additional research is needed to fully elucidate the mechanisms underlying the observed effects, particularly given the limitations of this study. First, we relied on hematoxylin and eosin staining to assess inflammation and re-epithelialization, which, while useful for structural evaluation, does not provide detailed insights. The inclusion of immunohistochemical markers would enhance the understanding of the healing process. Additionally, the limited number of experimental animals, although ethically justified, limits our findings. Moreover, this study did not include chemical analysis of the silkworm extracts, as it represents an initial screening within a broader systematic investigation conducted in our laboratory [24]. Finally, the absence of a mechanistic approach, particularly regarding molecular pathways involved in burn healing, remains a limitation. Future studies addressing these aspects shall provide a more comprehensive understanding of the therapeutic potential of *Bombyx mori* L. derivatives.

## 5. Conclusions

The cocoon and the silkworm body extract presented the best overall picture, demonstrating considerable effectiveness. The concentration of the silkworm body extracts appears to be proportional to the efficacy of the body extract. Gland’s extracts did not exhibit any noteworthy action. The combination of the gel with 60% body extract and cocoon showed slightly improved action compared to individual treatments.

This suggests that both the cocoon and the silkworm body extract hold significant therapeutic potential in the context of burn healing, with their effectiveness possibly being dose dependent. The lack of significant activity from gland extracts indicates that not all components of the *Bombyx mori* contribute equally to wound healing, highlighting the importance of targeting specific silk moth derivatives for therapeutic use. Furthermore, the marginally enhanced efficacy of the combined treatments suggests that a synergistic effect may exist between the cocoon and body extract, offering a promising avenue for further research into optimal formulation strategies for burn treatment.

## Figures and Tables

**Table 1:** Treatment modalities and formulations for pilot and study parts of the experiment. The table presents the treatment groups and corresponding formulations used in the pilot and study parts of the experiment, with sample sizes of *n* = 5 for the pilot part and *n* = 7 for the study part. The treatments include a control (no treatment), an excipients gel, various concentrations of silkworm body and gland extract gels, and cocoon application. In the study part, an additional combination treatment of silkworm body extract (60% *w*/*w*) gel with cocoon is included.

Pilot Part		Second Part	
Treatment	Formulation	Treatment	Formulation
Treatment 1	No treatment	Treatment 10	No treatment
Treatment 2	Excipients gel	Treatment 11	Excipients gel
Treatment 3	Gel with silkworm body extract (20% *w*/*w*)	Treatment 12	Gel with silkworm body extract (60% *w*/*w*)
Treatment 4	Gel with silkworm body extract (40% *w*/*w*)	Treatment 13	Cocoon
Treatment 5	Gel with silkworm body extract (60% *w*/*w*)	Treatment 14	Gel with silkworm body extract (60% *w*/*w*) and cocoon
Treatment 6	Gel with silkworm gland extract (20% *w*/*w*)		
Treatment 7	Gel with silkworm gland extract (40% *w*/*w*)		
Treatment 8	Gel with silkworm gland extract (60% *w*/*w*)		
Treatment 9	Cocoon		

**Table 2:** Scoring criteria for histopathological evaluation of tissue samples. Each parameter was scored based on the degree or presence of specific histopathological features observed during evaluation. Inflammation, oedema, hyperkeratosis, and wound thickness are rated on a scale from 0 to 3, indicating absence, mild, moderate, or heavy/total involvement, respectively. Ulceration, necrosis, and parakeratosis are evaluated as either absent (0) or present (1).

Scoring Criteria for Histopathological Evaluation				
Inflammation	0 (absence)	1 (mild)	2 (moderate)	3 (heavy)
Oedema	0 (absence)	1 (mild)	2 (moderate)	3 (heavy)
Hyperkeratosis	0 (absence)	1 (mild)	2 (moderate)	3 (heavy)
Wound thickness	0 (absence)	1 (superficial)	2 (moderate)	3 (total)
Ulceration	0 (absence)	1 (presence)		
Necrosis	0 (absence)	1 (presence)		
Parakeratosis	0 (absence)	1 (presence)		

**Table 3:** Histopathological assessment scoring for burn wounds in the pilot part of the study. The table summarizes the histopathological scores for burn wounds in various treatment groups from the pilot part of the study. Each treatment was evaluated for inflammation, edema, hyperkeratosis, wound depth, ulceration, necrosis, and parakeratosis, with an overall score representing the extent of tissue damage. The control group showed severe inflammation, edema, and moderate hyperkeratosis, scoring a total of 13 points. In contrast, the cocoon group demonstrated mild inflammation and superficial tissue damage, with a total score of 5 points, indicating the least tissue damage.

Pilot Part	Inflammation	Oedema	Hyperkeratosis	Wound Depth	Ulceration	Necrosis	Parakeratosis	Score
Control	3	3	2	3	1	1	0	**13**
Excipients	3	3	3	3	0	0	0	**12**
Body extract 20%	2	2	2	3	0	0	0	**9**
Body extract 40%	2	2	1	3	0	0	0	**8**
Body extract 60%	2	2	1	3	0	0	0	**8**
Glands extract 20%	3	3	3	3	1	1	0	**14**
Glands extract 40%	3	3	3	3	1	1	0	**14**
Grands extract 60%	3	3	2	3	1	1	0	**13**
Cocoon	1	1	2	1	0	0	0	**5**

**Table 4:** Histopathological assessment scoring for burn wounds in the study part of the experiment. The table provides a summary of histopathological scores for burn wounds in various treatment groups from the study part of the experiment. Each group was evaluated based on inflammation, edema, hyperkeratosis, wound depth, ulceration, necrosis, and parakeratosis, with an overall score representing the extent of tissue damage. The control group displayed severe inflammation and moderate edema, scoring a total of 10 points. The excipient group exhibited severe edema and extensive inflammation, with a notable presence of inflammatory exudate and hemorrhagic blistering, resulting in a score of 12 points. The combination of cocoon and body extract showed the least tissue damage with a score of 7 points.

Second Part	Inflammation	Oedema	Hyperkeratosis	Wound Depth	Ulceration	Necrosis	Parakeratosis	Score
Control	3	2	1	3	1	0	0	**10**
Excipients	3	3	1	3	1	0	1	**12**
Body extract 60%	2	2	1	3	0	0	0	**8**
Cocoon	2	2	1	3	0	0	0	**8**
Cocoon and body extract 60%	2	2	1	2	0	0	0	**7**

## Data Availability

All study’s data are available from the corresponding author upon reasonable request.
